# Specific microRNAs as novel biomarkers for combination chemotherapy resistance detection of colon adenocarcinoma

**DOI:** 10.1186/s40001-015-0183-8

**Published:** 2015-12-02

**Authors:** Jinsong Hu, Ye Xu, Sanjun Cai

**Affiliations:** Department of Colorectal Surgery, Fudan University Shanghai Cancer Center, Department of Oncology, Shanghai Medical College, Fudan University, Shanghai, China; Department of Gastrointestinal Surgical, Shanghai East Hospital, Tongji University School of Medicine, Shanghai, China

**Keywords:** Colon cancer, MicroRNAs, Biomarker

## Abstract

**Background and objectives:**

Colon cancer is a frequently occurring primary malignant tumor. Chemotherapy can reduce the risk of local and distant relapse. Therefore, it is very important to find new biomarkers that can predict chemoresistance and help in individuate treatment decision.

**Design and setting:**

Retrospective analysis of 126 patients, who were treated at our department between June 2010 and December 2014.

**Patients and methods:**

In this study, we examined the expression levels of 1200 human miRNAs in colon cancer tissues, using laser capture microdissection and microRNA profiling arrays. A validation study was done to corroborate a subset of the results, including expression levels of miR-4299, miR-196b, miR-324-5p, miR-455-3p and miR-939, through analyzing stage IV colon adenocarcinoma tissues (not responding and responding to the chemotherapy) with laser capture microdissection and quantitative real-time PCR. We analyze the relationship between the expression levels of these miRNAs and the survival rate of colon cancer patients by Kaplan–Meier method.

**Results:**

We found that miR-4299 and -196b have significant diagnostic value in chemoresistant colon cancer. MiR-4299 yielded an AUC (the areas under the ROC curve) of 0.810 and miR-196b yielded an AUC of 0.726 in discriminating chemoresistant colon cancer from controls. Combined with ROC analyses of these 2 miRNAs revealed an elevated AUC of 0.877 with 71.4 % sensitivity and 95.5 % specificity in discriminating chemoresistant colon cancer. The low level of miR-4299 expression and the high level of -196b expression are significantly correlated with better survival of colon cancer patients.

**Discussion:**

These data suggest that miR-4299 and -196b have strong potential as novel biomarkers for chemoresistance detection of colon cancer.

## Background

Colon cancer is one of the most frequent cancers and a common cause of cancer-related deaths [1]. Due to the poor prognosis and distant invasion and migration, the overall incidence of colon cancer is approximately 5 % and the 5-year survival rate of colon cancer patients is very low [2]. In China, the morbidity and mortality of colon cancer are 14.68/10^5^ and 6.66/10^5^, which is faster than the average increasing rate of the world. In Shanghai, the incidence of colon cancer is one of the highest in china (25.55/10^5^) [3]. Thus, the identification of new targets for the development of predicted chemoresistance is urgent in colon cancer patients.

MicroRNAs (miRNAs) are small, noncoding sequences, approximately 18–25 nucleotides long, which are post-transcriptional regulators of gene expression. They use imperfect base-pairing with the 3′-untranslated region (3′ UTR) of messenger (m) RNA, usually resulting in translational repression and gene silencing. Aberrant expression of miRNAs is implicated in numerous diseases and miRNA misregulation is also linked to many types of cancer [4, 5]. Depending on the genes miRNAs are regulated; miRNAs can function as either oncogenes or tumor suppressors [6].

After the discovery, that the miRNAs are involved in a significant number of biological processes, such as development, differentiation, apoptosis and proliferation, it became evident that miRNAs are widely involved in the initiation and progression of cancer. [7, 8] MicroRNAs are able to control protein expression. It has been suggested that modification of miRNA gene expression could be an important factor in the response to drug treatment [9].

The median progression-free survival of advanced colorectal cancer (CRC) patients has increased since the introduction of oxaliplatin in combination with 5-fluorouracilin systemic chemotherapy treatment [10]. However, colorectal cancer is resistant to chemotherapy and chemotherapy has been less effective against transforming cancerous cells in many patients, ultimately leading to the death of patients.

In a clinical context, Hansen found low expression of miR-126 in vessel structures of primary tumors associated with poor response to first-line XELOX therapy [11]. Further knowledge about the molecular mechanisms underpinning colon cancer chemoresistance may provide insights for better therapeutic opportunities to treat colon cancer patients. The aim of this study is to identify miRNAs that might be diagnostic and prognostic for chemoresistant colon cancer in relation to first-line treatment with capecitabine and oxaliplatin (XELOX).

## Methods

### Patients

The patients were recruited in this study. Patients were excluded from the study if they had a clinical diagnosis of hereditary nonpolyposis colon cancer, or if they had undergone chemotherapy or radiotherapy for a diagnosis of cancer at any site and any time before surgery. All patients underwent colorectal resection; however, at the same time the metastases (liver or lung) were not surgically removed. Tumors were staged according to the tumor node metastasis (TNM) staging system of the Union for International Cancer Control (UICC). The study included 126 patients with colon cancer histologically verified at the department of clinical pathology, Shanghai Cancer Center (Table 1). The patients underwent surgery in the period from June 2010 to December 2014. After surgery they received first-line chemotherapy. Pre-treatment examination consisted of standard hematologic parameters (blood routine, hepatic function, renal function, electrolytes and so on) and a CT scan of the chest and abdomen and ultrasound of the abdomen. All patients received a minimum three cycles of XELOX.

**Table 1:** Clinicopathological factors

Factors	Non-responders	%	Responders	%	*P* value
	*n* = 62		*n* = 64		
	Number		Number		
Age [mean (SD)]	54.3 (9.1)		57.0 (12.5)		0.713
Sex					0.472
Male	34	54.8	31	48.4	
Female	28	45.2	33	51.6	
Histology					
Adenocarcinoma	All		All		
Histological grade					0.485
Well	No		No		
Moderately	32	51.6	37	57.8	
Poorly	30	48.4	27	42.2	
Depth of tumor invasion					
ss, se, si	All		All		
Lymph node metastasis					0.292
≤3	33	53.2	40	62.5	
>3	29	46.8	24	37.5	
Venous invasion					0.383
Absent	35	56.5	41	64.1	
Present	27	43.5	23	35.9	
Nerve invasive					0.001*
Absent	39	62.9	21	32.8	
Present	23	37.1	43	67.2	
Liver metastasis					0.637
Absent	17	27.4	20	31.3	
Present	45	72.6	44	68.7	
Lung metastasis					0.248
Absent	50	80.6	46	71.9	
Present	12	19.4	18	28.1	
Tumor location					0.072
Distal colon	38	61.3	29	45.3	
Proximal colon	24	38.7	35	54.7	

subserosa (ss), penetration of serosa (se), and invasion of adjacent

structures (si), * P < 0.05

All patients received the same treatment (XELOX), which consisted of a 2-h intravenous infusion of oxaliplatin 130 mg/m^2^ on day 1 followed by oral capecitabine 1000 mg/m^2^ twice daily on days 1–14 (28 doses) of a 21-days cycle. Treatment was continued until disease progression or unacceptable toxicity.

Informed consent was obtained from all participants for the use of their tissue samples in this study. This project was approved by the Clinical Research Ethics Committee of Fudan University Cancer Hospital.

### Clinical evaluation of response criteria

Treatment response, according to RECIST (version 1. 0), was assessed every 9 weeks with clinical and radiologic examination using CT scan of the chest and abdomen (90 patients). Responding patients were classified as having either complete response (CR) or partial response (PR). Patients with stable disease (SD) or progressive disease (PD) were defined as non-responders. All CT scans were evaluated by the same investigator.

### Tissue processing

Fresh biopsies of colon tumors were collected between June 2010 and December 2014 during staging surgery (colon resection) at Shanghai Cancer Center, frozen within 5 min in liquid nitrogen, and stored at −80 °C. Investigators performing microRNA analysis were blinded to the clinical outcome of patients.

### Laser capture microdissection, RNA extraction and miRNA expression profiling

Colon cancer tissues were collected by laser capture microdissection (LCM) as previously described [12]. Briefly, colon cancer tissues were dissected in 1 × 0.5 cm, and frozen in optimal cutting temperature compound (OCT) with 25 % sucrose. Sections were cut at 10 μm, mounted onto slides with charged PEN-foil membranes (Leica Microsystems, Deerfield, IL, USA), fixed in 100 % ice-cold ethanol for 10 min, stained with hematoxylin for 1 min, and dehydrated in 100 % ethanol for 30 s and xylene for 5 min. Tissues of colon cell layer were isolated separately using an LMD6000 laser capture microdissection microscope (Leica Microsystems).

Total RNA isolation was performed using mirvana miRNA Isolation kit (Ambion, USA) according to the manufacturer’s protocol. RNA concentrations were measured using the NanoDrop ND-1000 spectrophotometer (Wilmington, USA). MiRNA expression profiles of CRCs were determined with Agilent human miRNA v162.0 (Santa Clara, USA). Expression levels were normalized against endogenous U6 control. Each slide was hybridized with 100 ng Cy3-labeled RNA using miRNA complete Labeling and Hyb kit (Santa Clara, USA) according to the manufacturer’s instructions. After hybridization, slides were washed in staining dishes (Thermo Shandon, USA) with Gene Expression Wash Buffer Kit (Santa Clara, USA). Slides were scanned by Agilent Microarry Scanner (Santa Clara, USA) and Feature Extraction software 10.7 (Santa Clara, USA) with default settings. Raw data were normalized by Quantile algorithm, Gene Spring Software 11.0 (Santa Clara, USA).

### Real-time PCR quantification of the miRNA

For the individual assay, the expression levels of mature miRNAs were determined by real-time quantitative polymerase chain reaction (q-PCR). Reverse transcription (RT) followed by real-time PCR amplification with Universal cDNA synthesis kit (EXIQON, Denmark). The experimental procedures were done according to the protocol recommended by the manufacturer. Quantitative real-time PCR was performed using the Power SYBR Green Master Mix (EXIQON, Denmark) on the 7900HT Real-time PCR System (Applied Biosystems). All data values were normalized by Human U6. The threshold cycle data were determined using the default threshold settings. All real-time PCR reactions were run in triplicate and average *C*_t_ and SD values were calculated.

### Statistical analysis

Clinicopathological factors were compared using *X*^2^ test or *T* test. Expression levels of tissue miRNAs were compared using the Mann–Whitney *U* test or the Kruskall–Wallis test. Receiver operating characteristics (ROC) curves were established to evaluate the diagnostic value of tissue miRNAs for differentiating between non-responder and responder. Overall survival was estimated using the Kaplan–Meier method. A *p* value of less than 0.05 was considered statistically significant. All statistical analysis was performed with SPSS 19.0 software (SPSS, Inc., Chicago, IL, USA).

## Results

### Patient population

A total of 126 participants including 62 colon cancer patients of non-responders and 64 colon cancer patients of responders were recruited into this study (Table 1). No significant differences of age, gender, histological grade, lymph node metastasis, venous invasion, liver metastasis, lungs metastasis and tumor location were found between non-responder and responder, but there were significant differences of nerve invasive in the colon cancer(P = 0.001). All patients were followed up until death or the end of the study.

### Expression profiles of miRNAs correlated to colon cancer chemoresistance

**Fig. 1:**
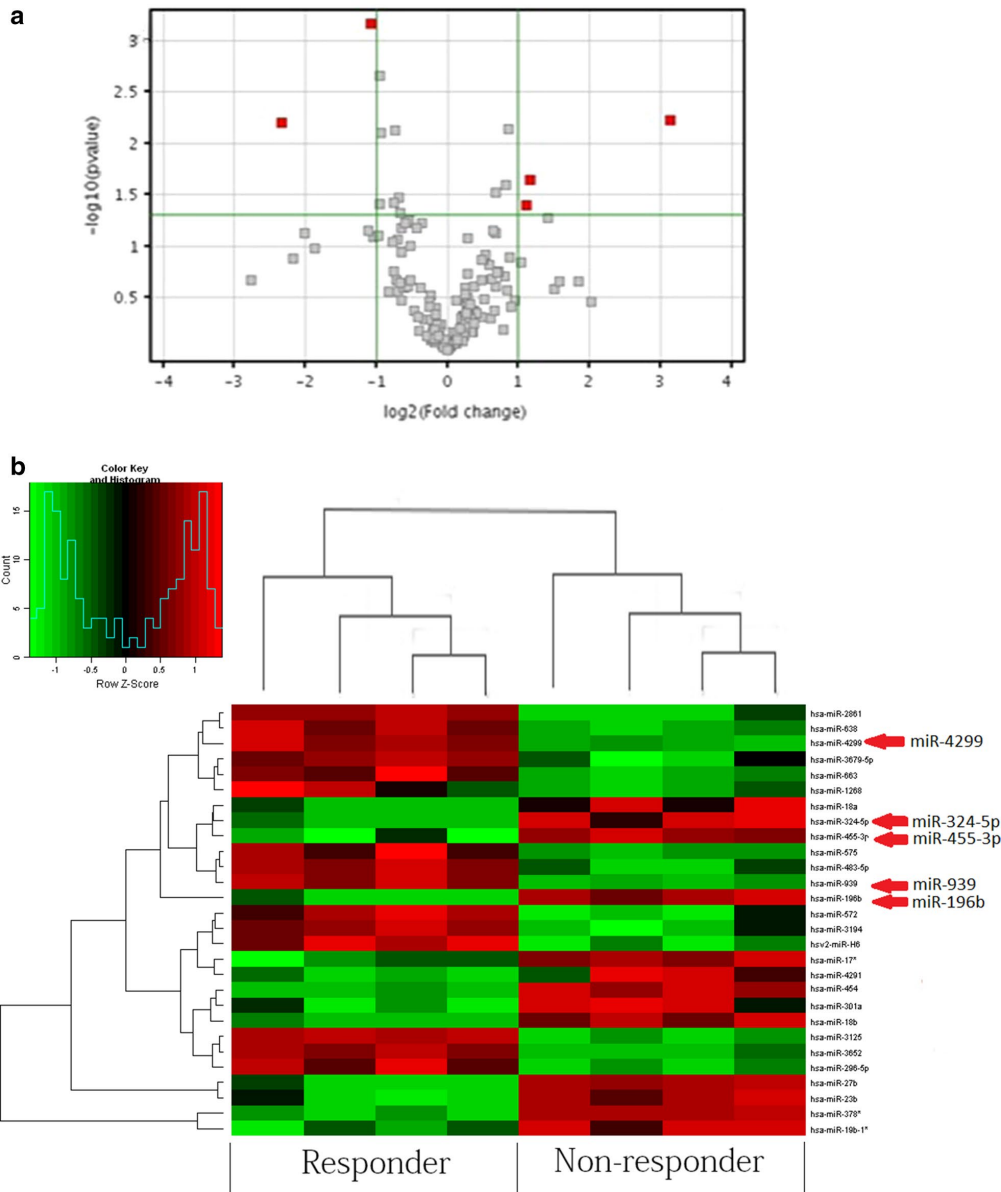
Dysregulation of miRNA in human colon cancer. The miRNA profiling analysis on 4 pairs of colon cancer tissue. miRNA expression was normalized against endogenous control U6. **a** Volcano plot illustrating the biological and statistical significances of differential miRNA expressions between non-responders and responders in human colon cancer. miRNAs that were significantly expressed in human colon cancer are marked in *red*. **b** Heat map diagram generated by unsupervised clustering analysis with 5 significantly dysregulated miRNAs in their human colon cancers. miRNA expression profile effectively segregated non-responder samples from their corresponding responder samples

To investigate the roles of miRNA in colon cancer chemoresistance, we first examined the miRNA expression profiles with Agilent Human MicroRNA Array (Among the 1367 unique miRNA probes) on 4 responder colon cancer and 4 non-responder colon cancer to the chemotherapy. After normalization with the endogenous control U6, we found that 5 miRNAs were significantly dysregulated in responder as compared with non-responder (P < 0.05, paired t test) (Fig. 1a, b; Table 2). The miRNA profiling data indicate that miRNAs frequently were dysregulated in human colon cancer. Comparing among the group between non-responder and responder, miR-4299 and -939 were the most up-regulated and miR-196b, miR-324-5p and 455-3p were the most down-regulated miRNAs. We therefore need to further investigate 5 miRNAs.

**Table 2:** Significant dysregulation of miRNAs in chemoresistant colon cancer

miRNAs	*P*	Mean fold change
hsa-miR-196b	0.0059	8.502
hsa-miR-324-5p	0.0224	2.280
hsa-miR-455-3p	0.0391	2.082
hsa-miR-4299	0.0062	0.191
hsa-miR-939	0.0007	0.472

*hsa* human

### Differential overexpression of miR-4299 and -196b in the colon cancer

**Fig. 2:**
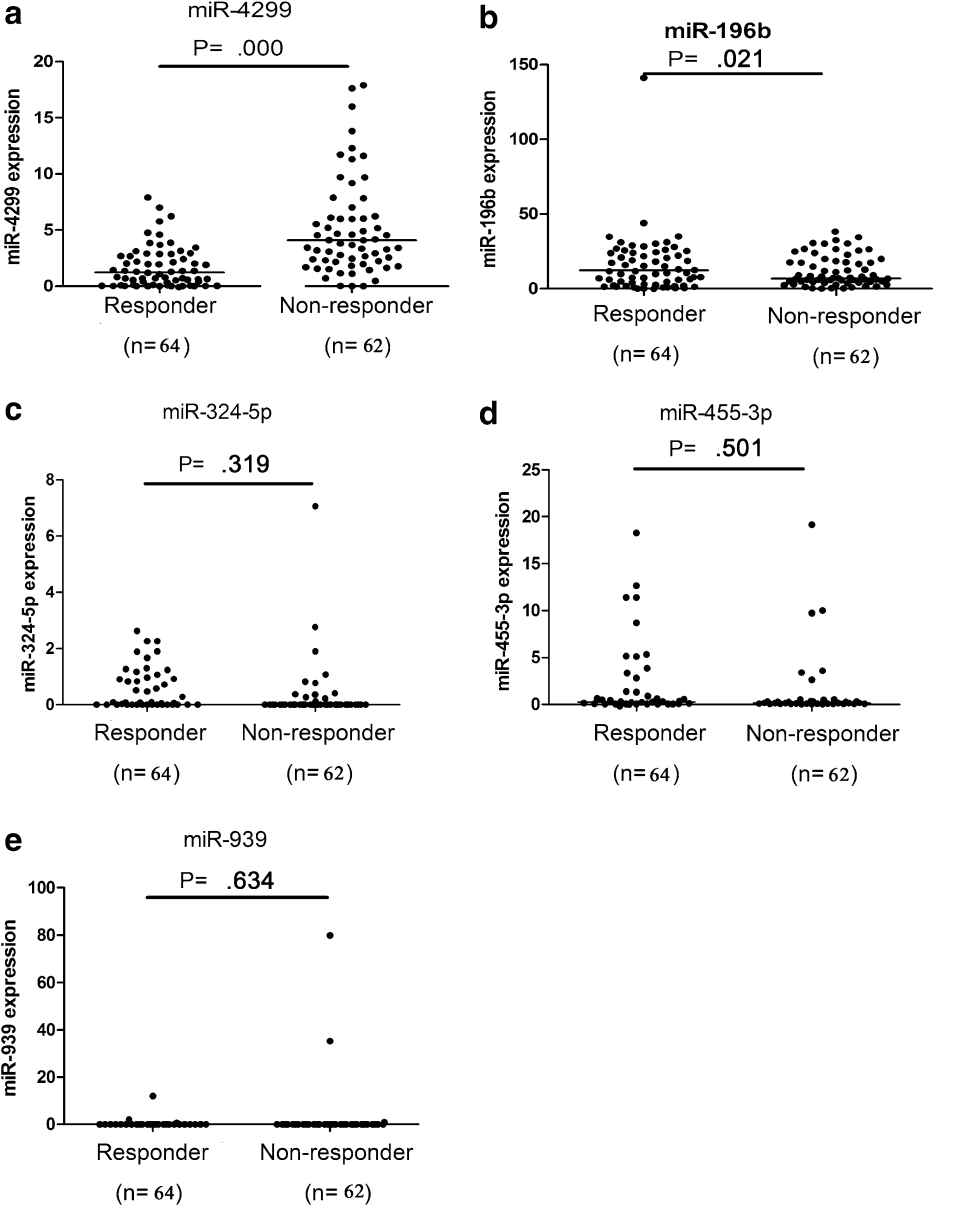
*Dots* indicate of the relative quantification (RQ) values of miRNA expression levels (**a** miR-4299, **b** miR-196b, **c** miR-324-5p, **d** miR-455-3p, **e** miR-939), normalized by U6. The line represents the median value. Mann–Whitney *U* test was used to determine statistical significance

To evaluate the correlation between these candidates, we examined the expression of miR-196b, -4299, -324-5p, -455-3p and -939 in a panel of 126 malignant colon cancer tissues, including 62 non-responders and 64 responders, by individual real-time PCR. MiR-4299 and -196b were significantly non-responder relative to responder colon cancer patient (*P* < 0.001 and *P* = 0.021), when compared with the U6 control, as shown in Fig. 2. No significant difference was observed in the levels of miR-324-5p, -455-3p and -939 between non-responder colon cancer patients and controls (*P* = 0.319 for miR-324-5p, *P* = 0.501 for miR-455-3p, *P* = 0.634 for miR-939). Taken together, our results determined that expression levels of miR-4299 were definitively higher in non-responder compared with responder and the expression levels of miR-196b were definitively lower. This suggests that they could be relevant to the chemoresistance of colon cancer.

### The diagnostic value of miR-4299 and -196b for the chemoresistance of colon cancer

**Fig. 3:**
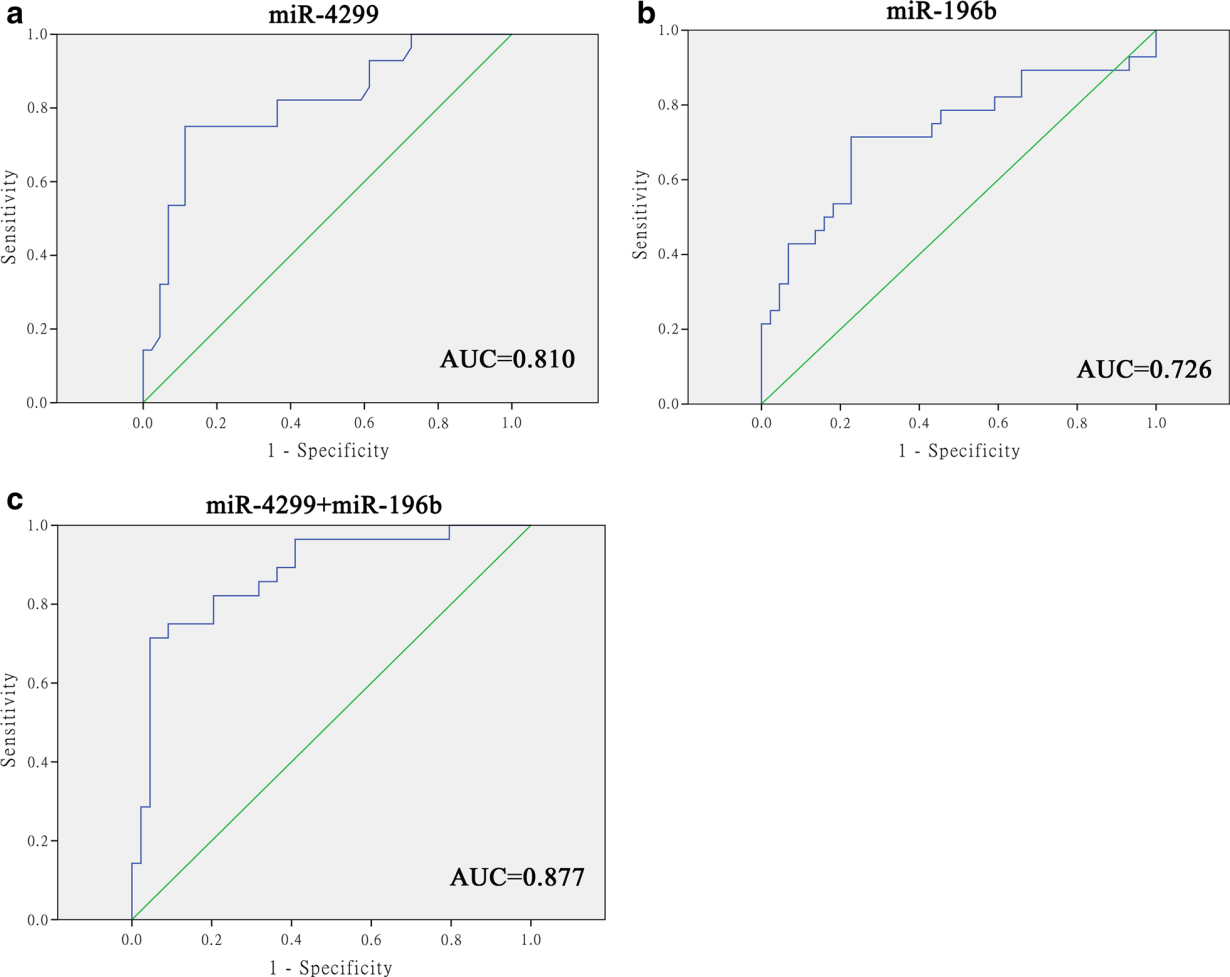
Receiver operating characteristics (ROC) curve analysis using miR-4299 and miR-196b for discriminating chemoresistance of colon tumors. miR-4299 yielded an AUC (the areas under the ROC curve) of 0.810 (95 % CI 0.704–0.917) with 75.0 % sensitivity and 88.6 % specificity in discriminating non-responder (**a**), and miR-196b yielded AUC of 0.726 for miR-196b (95 % CI 0.597–0.856) with 71.4 % sensitivity and 77.3 % specificity in discriminating non-responder (**b**). Combined ROC analyses revealed an elevated AUC of 0.877 (95 % CI 0.793–0.962) with 71.4 % sensitivity and 95.5 % specificity in discriminating non-responder (**c**)

To test the diagnostic value of miR-4299 and -196b in the chemoresistance of colon cancer, we measured the expression of these miRNAs in tissue samples of 62 non-responders, and found a significant expression when compared to the responders (p < 0.001 for miR-4299, P = 0.021 for miR-196b). ROC curve analyses also showed that both of the 2 miRNAs could differentiate non-responders from controls of responders with an AUC of 0.810 for miR-4299 (95 % CI 0.704–0.917) and 0.726 for miR-196b (95 % CI 0.597–0.856), respectively (Fig. 3a, b). At the cutoff value of 1.46 for miR-4299, the sensitivity and the specificity were 75.0 and 88.6 %, respectively. At the cutoff value of 9.54 for miR-196b, the sensitivity and the specificity were 71.4 and 77.3 %, respectively. Combination ROC analyses also revealed a little increased AUC value of 0.877 (95 % CI 0.793–0.962) with 71.4 % sensitivity and 95.5 % specificity (Fig. 3c).

### Under- and over-expression of miR-4299 and -196b expression correlated with better survival in stage IV colon cancer patients

**Fig. 4:**
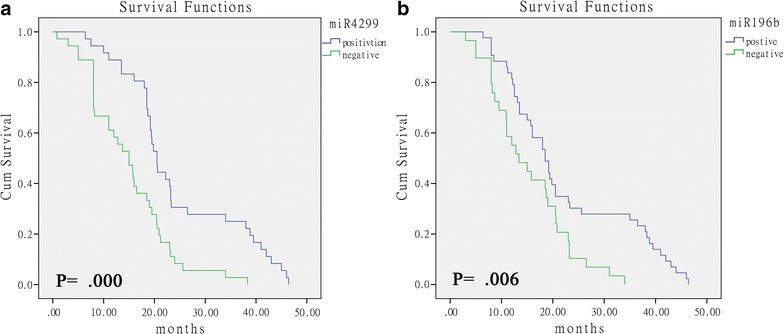
Kaplan–Meier curves depicting overall survival (OS) depending on the tumor miRNA expression. OS according miR-4299 expression (**a**). OS according miR-196b expression (**b**)

To evaluate the clinical implications of the miRNAs identified in this study, we analyzed the clinical data from 126 stage IV colon cancer patients (62 non-responders and 64 responders) whose samples were tested in the validation experiment. The correlation between expression levels of miRNAs and overall survival were measured through Kaplan–Meier survival curve analysis with comparison using a binomial variable of high or low expression relative to the cutoff values of the miRNAs in the ROC curve analyses. Statistically significant correlation was observed between the overall survival and the expression levels of miR-4299 and miR-196b (Fig. 4a, b; P = 0.000, P = 0.006). According to miR-4299 and miR-196b values, the median OS were 18.7 (miR-4299, CI 17.4–20.0) and 18.0 (miR-196a, CI 15.1–20.9). Collectively, the finding suggests that miR-4299 and miR-196b expression may play an important role in colon cancer chemotherapy prognosis.

## Discussion

Surgery is the most effective treatment for colon cancer. However, in locally advanced stages, radiotherapy and chemotherapy reduces the risk of local and distant relapse. Consequently, a multidisciplinary approach is currently the standard. complete pathologic disappearance of tumor cells after neoadjuvant chemotherapy occurs in about 20 % of patients and is correlated with a very good long-term prognosis. In the era of customized treatment, we should explore new identifiable predictive factors for conventional treatments, such as chemotherapy. The ultimate goal is to select the most effective and less toxic treatment for every patient.

Recent studies have implicated miRNAs in a variety of human cancers, and their expression signatures can provide insight into the diagnosis and prognosis of human cancers [13–20]. At first, this study provides the report of quantitative global miRNA expression profiles including expression data for 1200 human miRNAs in stage IV colon adenocarcinoma from clinical tissue samples. Then these tissue samples were mated to sex, age, histology, histological grade, tumor location and metastasis location. It has been reported that miRNA expression profiles reflect cellular differentiation and distinguish tumors of different developmental origin [21, 22]. Our study revealed significant differences between the expression levels of 5 miRNAs in the sets of 8 non-responders and control. Secondly, in most of miRNAs we identified (miR-196b, miR-4299, miR-324-5p, miR-455-3p and miR-939), the potential linkage between their expression and chemoresistance was already established; then some of them were identified for the first time. Furthermore, we showed that miR-4299 and -196b are strongly regulated in non-responder colon cancer. Therefore, the mechanisms obtained in this study need to be further validated in more detailed models and on an independent set of patients, before applying in clinical practice.

In this study, we found that the levels of miR-4299 and -196b in tissue samples from non-responders were significantly expressed than those in responders. Both miR-4299 and -196b had significant diagnostic value for colon cancer and yielded AUC of 0.810 and 0.726, respectively. Combined ROC analyses using these 2 targets could yield an increased AUC of 0.877 with 71.4 % sensitivity and 95.5 % specificity in discriminating non-responder from responder control. These suggest their potential value for early detection of chemoresistant colon cancer before we can initiate chemotherapy. To our knowledge, it is the first report to evaluate the diagnostic value of miRNAs on assessment of colon cancer resistant to first-line chemotherapy. The under- and over-expression of miR-4299 and -196b are significantly correlated with better survival of colon cancer patients.

Currently, miR-4299 is no more related research. MiR-196 is a negative regulator of several HOX genes. MiR-196 recognizes HOX mRNA with a nearly perfect match and cleaves the HOXB8 mRNA directly [23]. The importance of HOX gene expression for A-P patterning has been well characterized in developing limbs and the central axis [24]. Therefore, elevation of the expression of miR-196 in human cells may correlate with several phenotypes observed during developmental stages. MiR-196b can be assigned with the tumor suppressor function in acute lymphoblastic leukemia [25, 26]. Plummer reported miR-196b is responsive to vascular endothelial growth factor (VEGF) stimulation, thus miR-196b may constitute a novel strategy for inhibiting tumor angiogenesis [27]. Popovic reported that miR-196b function is necessary for MLL fusion-mediated immortalization and that overexpression of miR-196b increases the proliferation and survival of normal bone marrow hematopoietic progenitor cells [28]. Tsaithe et al. reported that high frequency of miR-196b promoter hypomethylation found in gastric cancer, miR-196b overexpression could provide a useful tumor marker [29]. Guan results suggest that miR-196b may play a role in the malignant progression of gliomas and may be a prognostic predictor in glioblastomas [30]. Lakomy reported miR-196b to be associated with survival of glioblastoma multiforme. MiR-196b was shown a positive correlation with OS [31].

In conclusion, miR-4299 and -196b appear to be novel biomarkers for detection of chemoresistant colon cancer. Our data serve as basis for further investigation, preferably in large prospective studies before these 2 miRNAs can be used as a screening tool for the treatment of colon cancer in routine clinical practice.

## Abbreviations

miRNAs: microRNAs
XELOX: capecitabine and oxaliplatin
TNM: tumor node metastasis
CR: complete response
PR: partial response
SD: stable disease
PD: progressive disease
